# Variação anatômica venosa rara em membros inferiores

**DOI:** 10.1590/1677-5449.007216

**Published:** 2016

**Authors:** Melissa Andreia de Moraes Silva, Hanna Fatima Paranaíba Mesquita, Iara Gabriel Carneiro, Arturo Eduardo Krupa, Seleno Glauber de Jesus Silva, Rodolfo Souza Cardoso

**Affiliations:** 1 Faculdade de Medicina de Itajubá – FMIt, Cirurgia Vascular, Itajubá, MG, Brasil.

**Keywords:** veia femoral, veia poplítea, anormalidades congênitas, anatomia, variação anatômica

## Abstract

A anatomia do sistema venoso dos membros inferiores é uma das mais complexas no corpo humano. Devido a essa condição, é de extrema importância saber identificar variações que possam acometê-la, como as malformações congênitas. Em casos de agenesia de veias profundas, como uma malformação vascular rara, o quadro clínico pode manifestar-se com insuficiência venosa crônica, que pode evoluir com edema, hiperpigmentação e úlcera de membro inferior. Assim, em muitos casos, torna-se uma doença incapacitante e de difícil tratamento. Apresenta-se um caso de agenesia de segmento venoso femoropoplíteo no membro inferior direito em paciente de 36 anos de idade, que cursou com edema e varizes de grosso calibre no membro acometido.

## INTRODUÇÃO

A anatomia venosa dos membros inferiores é muito variável devido a malformações venosas que ocorrem durante o desenvolvimento tardio do embrião, principalmente na fase final da embriogênese^1^. Acredita-se que um defeito generalizado no mesoderma pode levar a alterações vasculares, como a agenesia de veias profundas. A malformação venosa é a alteração vascular congênita mais comum, geralmente se apresentando como uma única lesão^2^. Porém, em 15-20% dos casos, ela pode se apresentar como uma lesão mista, combinada com outras malformações vasculares congênitas, como as malformações linfáticas e arteriovenosas^3^.

A veia femoral pode ser duplicada parcialmente ou em toda a sua extensão. Ocasionalmente, passa através do canal dos adutores, acima da artéria femoral, permanecendo paralela à essa até a união à veia profunda, onde forma a veia femoral comum^4^. Em um estudo realizado sobre as malformações no segmento venoso femoropoplíteo, foram descritas quatro categorias distintas de variações: (1) agenesia em 0,3% dos casos; (2) multiplicação, sendo isolada da veia femoral em 21% dos casos, isolada da veia poplítea em 2% e em ambas em 6%; (3) variação anatômica do curso venoso em 8%; e (4) união alta das veias tibiais, evidenciada em 7% dos casos. A principal variação é vista na veia femoral, onde 6-46% dos pacientes apresentaram duplicação ou múltiplos vasos. Nesse mesmo estudo, a incidência de agenesia do segmento venoso femoropoplíteo foi de 0,2% em membro inferior direito (MID), 0,4% em membro inferior esquerdo (MIE), e nenhum caso em ambos os membros simultaneamente^5^.

Em casos de agenesia de veias profundas, o quadro clínico pode manifestar-se com insuficiência venosa crônica (IVC), que é uma síndrome clínica composta por varizes de membros inferiores, dermatoesclerose, edema, hiperpigmentação (dermatite ocre) e úlcera de membro inferior, tornando-se, muitas vezes, uma doença incapacitante e de difícil tratamento. As principais causas de IVC são a incompetência de veias perfurantes, superficiais e/ou profundas, o que caracteriza a forma primária da doença; e casos de obstrução venosa proximal, fístulas arteriovenosas, disfunção da musculatura da panturrilha e malformações venosas congênitas, que ilustram as causas secundárias de IVC^6^.

O diagnóstico de uma malformação venosa, muitas vezes, pode ser feito com uma história cuidadosa e exame físico. Com frequência, o estudo invasivo com flebografia não é necessário para o diagnóstico de rotina, mas é essencial para o planejamento e tratamento terapêutico^7^. Além disso, a avaliação da linha de base inicial deve incluir uma busca ativa do examinador pelas complicações agudas relacionadas às malformações venosas, como trombose venosa superficial e profunda e embolia pulmonar, e também pelas complicações crônicas e sequelas, como desvios na marcha e escoliose com inclinação pélvica, que são alterações associadas comuns. Confirmado o diagnóstico de IVC causada provavelmente por agenesia de veia profunda, o tratamento é baseado na mudança de hábitos, uso de meia compressiva e medicamentos, uma vez que a cirurgia é contraindicada nesse caso^8^.

O objetivo deste trabalho é descrever um caso raro e pouco descrito na literatura de um paciente portador de IVC provavelmente causada pela agenesia de segmento venoso femoropoplíteo no MID, que evoluiu com formação de varizes de grosso calibre no membro acometido. Este trabalho foi aprovado pelo CEP da Plataforma Brasil, em 2015, sob o número do parecer 1.361.567.

## DESCRIÇÃO DO CASO

Paciente MJB, sexo masculino, 36 anos, branco, auxiliar de produção. Compareceu à consulta no dia 24/05/2014 com queixa de varizes em MID, associadas a edema assimétrico há 10 anos, com piora progressiva. Negou prurido, lesões de pele e trauma prévio ao atendimento. Não apresentava história de evento tromboembólico, cirurgia venosa, outras comorbidades ou história familiar de doença venosa.

**Figura 1:**
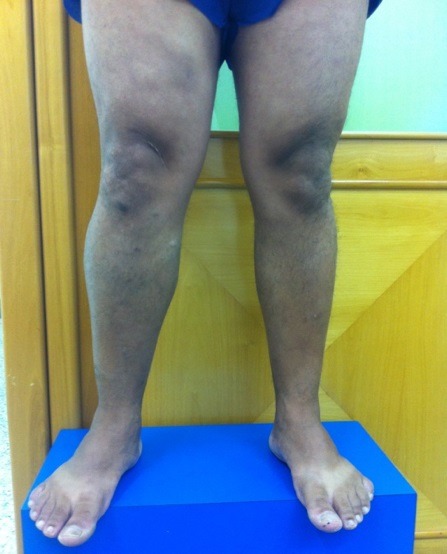
Membros inferiores com paciente em posição ortostática, vista frontal, evidenciando assimetria do membro inferior direito.

**Figura 2:**
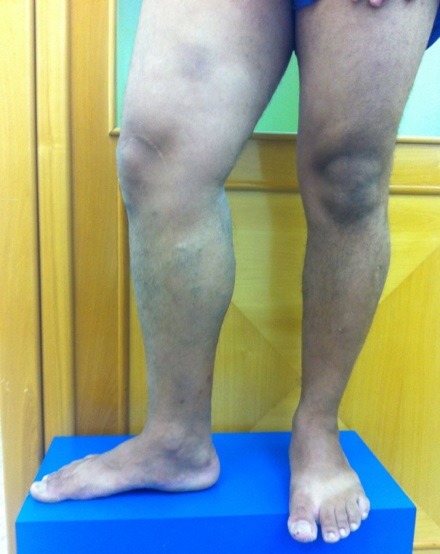
Membros inferiores com paciente em posição ortostática, vista frontal, evidenciando assimetria do membro inferior direito.

Ao exame clínico, apresentou veias tortuosas de grosso calibre em MID (Figuras 1, 2 e 3) e edema leve (+/4+). Não foram observadas lesões de pele ativas ou cicatriciais.

**Figura 3:**
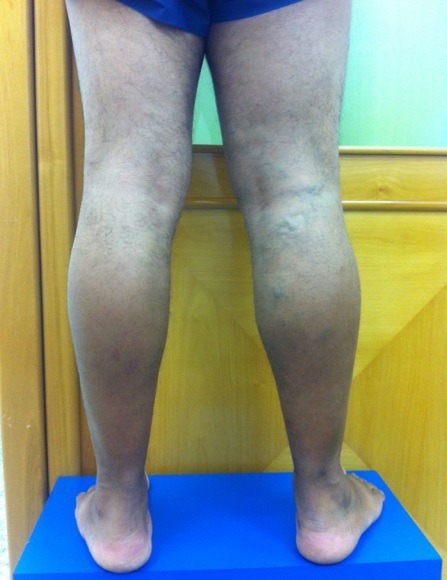
Membros inferiores com paciente em posição ortostática, vista dorsal, evidenciando varizes em perna proximal.

**Figura 4:**
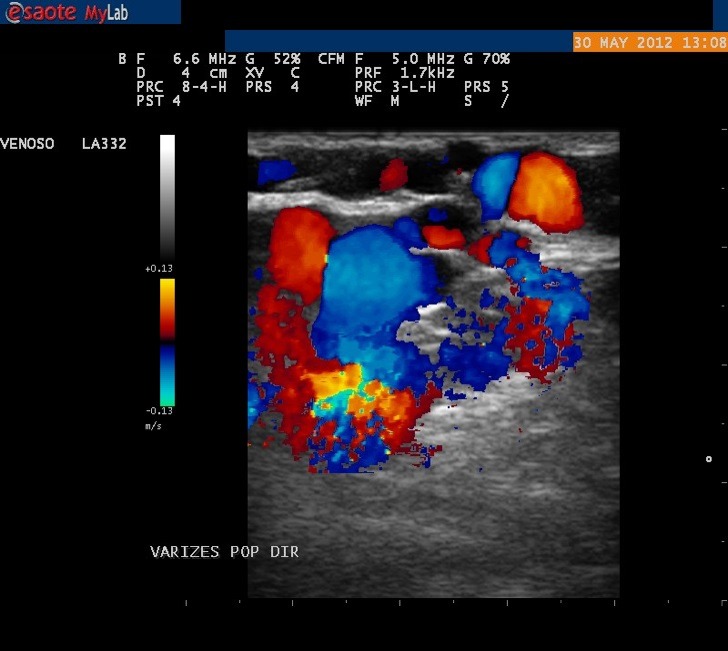
Ultrassom Doppler evidenciando varizes com distribuição atípica em membro inferior direito.

**Figura 5:**
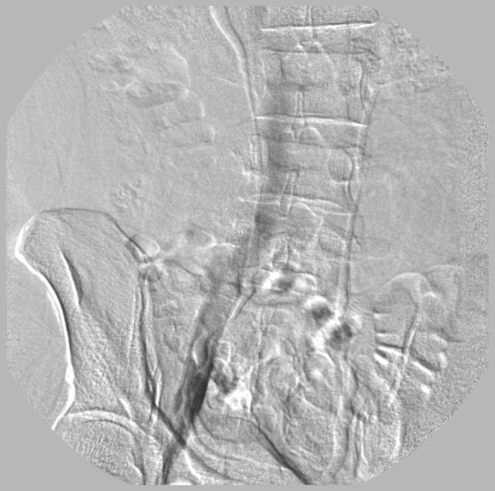
Flebografia ascendente mostrando veias ilíacas e cava inferior sem alterações.

**Figura 6:**
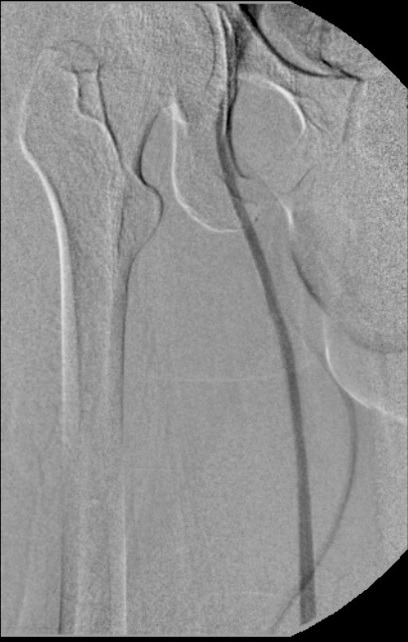
Flebografia ascendente mostrando veia safena magna e veia femoral comum. Não foi observada a veia femoral neste segmento.

Foi solicitado um ultrassom vascular com Doppler de membros inferiores, no qual não foram visibilizadas as veias poplítea e femoral no MID, com veias safena magna e parva presentes, sem sinais de refluxo valvar (Figura 4). Na sequência da investigação, o paciente foi submetido a flebografia do MID, onde foi confirmada a ausência das veias femoral e poplítea (Figuras 5, 6 e 7).

**Figura 7:**
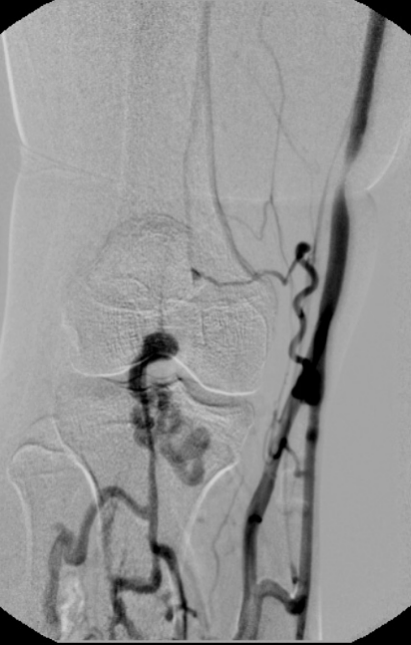
Flebografia ascendente mostrando veias profundas de perna com drenagem anômala para veia safena magna. Não foi visualizada a veia poplítea.

Após confirmação do diagnóstico, foi indicado ao paciente o uso de meias elásticas e medicação paliativa para controle das varizes. Mesmo com inúmeras tentativas de contato para seguimento do caso, o paciente não compareceu à consulta de retorno para avaliação da sua evolução.

## DISCUSSÃO

As anormalidades anatômicas do sistema venoso profundo são uma entidade rara causada por distúrbios no desenvolvimento embrionário, normalmente ao final da embriogênese^5^. Em muitos casos, o diagnóstico é difícil e o paciente permanece por longos períodos com tratamento errôneo de outra doença vascular, que pode apresentar sintomas semelhantes aos de anomalias anatômicas. O exame físico fornece ferramentas para a construção do diagnóstico de IVC, mas não é suficiente para detectar as estruturas envolvidas e a extensão da lesão. Com isso, o exame inicial da investigação, o ultrassom vascular com Doppler, que foi solicitado neste caso, oferece análise anatômica e hemodinâmica das estruturas vasculares envolvidas, auxiliando no diagnóstico e na diferenciação dos tipos de malformações^9^. O exame evidenciou uma provável agenesia de sistema venoso profundo no MID, que foi confirmada pela flebografia.

Em estudo retrospectivo realizado com 445 pacientes (890 membros), que analisou a anatomia venosa com angiotomografia venosa de pacientes com queixas de varizes e edema, evidenciou-se o achado de agenesia do segmento venoso profundo femoropoplíteo, o mesmo relatado no presente estudo, em apenas 0,3% dos casos (três membros)^5^.

Essa condição é umas das causas de IVC, que atualmente é uma das mais frequentes doenças que acomete tanto a população brasileira quanto a mundial. Por se tratar de uma doença crônica e evolutiva, cerca de 3-11% dos pacientes portadores de varizes poderão chegar a estágios mais avançados da doença com complicações irreversíveis na pele^10^. No caso descrito, o paciente apresentava dor em queimação acompanhada de edema no membro acometido, mas não apresentava sinais clínicos sugestíveis de processo grave.

A clínica é variável, incluindo desde pacientes assintomáticos até casos graves de úlcera venosa^11^. No caso relatado, o paciente apresentava sintomas moderados da doença de forma unilateral, com queixa de edema e presença de varizes de grosso calibre.

Quase todas as malformações vasculares se beneficiam com tratamento compressivo, adequado, monitorizado e iniciado desde cedo, que evita uma piora progressiva do quadro^12^. Nos casos mais raros em que as varizes são causadas por agenesia venosa congênita, pode-se erroneamente inferir que o sistema venoso profundo é pérvio e que se trata apenas de insuficiência venosa primária. Esses casos são problemáticos no que diz respeito ao tratamento cirúrgico. Se for utilizada uma estratégia pouco apropriada, com retirada das veias varicosas superficiais, poderá ocorrer agravamento do quadro, com sobrecarga do sistema venoso superficial restante, sendo que se deve lembrar que o sistema venoso profundo é incompleto^13^.

Portanto, em casos de hipoplasia ou agenesia do sistema venoso profundo, é contraindicado o tratamento cirúrgico das varizes. São mais adequados a mudança de hábitos, o uso de meias elásticas e a medicação paliativa^14^, como foi a indicação para o paciente deste caso.

## CONCLUSÃO

A anatomia venosa dos membros inferiores é muito variável; porém, a agenesia do sistema venoso profundo é rara. O diagnóstico precoce nem sempre ocorre, e a escolha terapêutica correta nesses casos é de fundamental importância, devendo ser mantida em pilares conservadores para evitar a ressecção das veias superficiais desses membros.
