# Supplementary material for: Variação anatômica venosa rara em membros inferiores
Source: J Vasc Bras. 2016 Oct-Dec;15(4):334–8. [Article in Portuguese] doi: 10.1590/1677-5449.007216 (PMC5829735; doi:10.1590/1677-5449.007216)
Supplement: Translation

# Translation: A rare venous anatomic variation of the lower limb

**Authors:** Melissa Andreia de Moraes Silva, Hanna Fatima Paranaíba Mesquita, Iara Gabriel Carneiro, Arturo Eduardo Krupa, Seleno Glauber de Jesus Silva, Rodolfo Souza Cardoso

**Affiliations:** 1 Faculdade de Medicina de Itajubá – FMIt, Cirurgia Vascular, Itajubá, MG, Brazil.

**Keywords:** femoral vein, popliteal vein, congenital abnormalities, anatomy, anatomic variant

## Abstract

The anatomy of the venous system of the lower limbs is among the most complex in the human body. In view of this, it is extremely important to know how to identify variations that can affect it, such as congenital malformations, for example. In cases of a rare vascular malformation such as agenesis of deep veins, clinical status may manifest with chronic venous insufficiency, which can progress with edema, hyperpigmentation, and lower limb ulcers. This is very often therefore an incapacitating disease that is difficult to treat. This article describes a case of agenesis of a segment of femoropopliteal vein in the right lower limb of a 36-year-old patient who had edema and large caliber varicose veins in the affected limb.

## INTRODUCTION

The venous anatomy of the lower limbs is highly variable because of venous malformations that occur during later development of the embryo, particularly during the final phase of embryogenesis.^1^ It is believed that a generalized defect of the mesoderm can cause vascular abnormalities, such as agenesis of deep veins. Venous malformation is the most common congenital vascular disorder and is generally a single lesion.^2^ However, in 15-20% of cases it will present as a mixed lesion, combined with other congenital vascular malformations, such as lymphatic and arteriovenous malformations.^3^

The femoral vein may be duplicated partially or throughout its entire length. Occasionally it will pass through the adductor canal, above the femoral artery, running parallel to it until it joins the deep vein, forming the common femoral vein.^4^ A study of malformations of the femoropopliteal venous segment described four distinct categories of variants: (1) agenesis, in 0.3% of cases; (2) multiplications, the femoral vein in isolation in 21% of cases, the popliteal vein in isolation in 2%, and both veins in 6%; (3) anatomic variant of the paths followed by veins, in 8%; and (4) high junction of the tibial veins, seen in 7% of cases. The principal variation was seen in the femoral vein, where 6-46% of the patients exhibited duplicated or multiple vessels. The same study reported an incidence of agenesis of the femoropopliteal venous segment of 0.2% in the right lower limb (RLL), 0.4% in the left lower limb (LLL), and no cases whatsoever in both lower limbs simultaneously.^5^

In cases of agenesis of deep veins, clinical status may manifest with chronic venous insufficiency (CVI), which is a clinical syndrome comprising varicose veins of lower limbs, dermatosclerosis, edema, hyperpigmentation (ochrodermatitis) and lower limb ulcers and is very often an incapacitating disease that is difficult to treat. The principal causes of CVI are incompetence of perforating, superficial, and/or deep veins, in the primary form of the disease; and proximal venous obstruction, arteriovenous fistulas, dysfunction of the musculature in the calf, and congenital venous malformations, which are all secondary causes of CVI.^6^

Venous malformations can often be diagnosed on the basis of a careful patient history and physical examination. Invasive examination with phlebography is often unnecessary for routine diagnosis, but it is essential for planning treatment.^7^ Furthermore, the initial baseline assessment should include an active search for acute complications related to venous malformations, such as superficial and deep venous thromboses and pulmonary embolisms, and for chronic complications and sequelae, such as problems walking and scoliosis with pelvic tilt, which are common associated disorders. Once a diagnosis of CVI probably caused by agenesis of a deep vein has been confirmed, treatment is founded on lifestyle changes, compressive stockings, and medications, since surgery is contraindicated in these cases.^8^

The objective of this study is to describe a rare case with few reports in the literature of a patient with CVI, probably caused by agenesis of a femoropopliteal venous segment in the RLL, who developed large caliber varicose veins in the affected limb. This project was approved by the Plataforma Brasil Ethics Committee in 2015, under hearing number 1.361.567.

## CASE DESCRIPTION

The patient (MJB), was a 36-year-old, white, male production assistant. He presented for a consultation on 24 May 2014 complaining of varicose veins in the RLL, associated with asymmetrical edema with onset 10 years previously and progressive deterioration. He stated that he was free from itching, skin lesions, and previous traumatisms. He had no history of thromboembolic events, venous surgery, or other comorbidities and had no family history of venous disease.

On clinical examination, he exhibited large caliber tortuous veins in the RLL (Figures 1, 2 and 3) and mild edema (+/4+). No active or healed lesions of the skin were observed.

**Figure 1:** Lower limbs with patient standing upright, front view, showing asymmetry of right lower limb.

**Figure 2:** Lower limbs with patient standing upright, front view, showing asymmetry of right lower limb.

**Figure 3:** Lower limbs with patient standing upright, rear view, showing varicose veins in proximal leg.

A vascular ultrasound examination of the lower limbs with Doppler was unable to detect the popliteal or femoral veins in the RLL, but showed the great and small saphenous veins, which did not have signs of valvular reflux (Figure 4). The investigation was continued with phlebography of the RLL, which confirmed the absence of the femoral and popliteal veins (Figures 5, 6, and 7).

**Figure 4:** Doppler ultrasound showing varicose veins with atypical distribution in the right lower limb.

**Figure 5:** Ascending phlebography showing iliac vein and inferior vena cava with no abnormalities.

**Figure 6:** Ascending phlebography showing great saphenous vein and common femoral vein. The femoral vein was not observed in this segment.

**Figure 7:** Ascending phlebography showing deep veins of the leg with anomalous drainage to the great saphenous vein. The popliteal vein was not visible.

After confirmation of the diagnosis, the patient was prescribed elastic stockings and palliative medication to control the varicose veins. Countless attempts were made to contact the patient for follow-up, but he never attended a follow-up consultation so his progress is unknown.

## DISCUSSION

Anatomic abnormalities of the deep vein system are a rare entity caused by disorders of embryonic development; normally at the end of embryogenesis.^5^ In many cases, diagnosis is difficult and patients remain for long periods being erroneously treated for other vascular diseases that can exhibit similar symptoms to anatomic anomalies. Physical examination offers the tools needed to construct a diagnosis of CVI, but is insufficient to detect the structures involved or the extent of lesions. The initial work-up examination is therefore vascular ultrasound with Doppler, as was employed in this case, offering anatomic and hemodynamic analysis of the vascular structures involved, aiding with diagnosis and differentiation of types of malformations.^9^ In this case, the examination revealed a probable agenesis of the deep vein system in the RLL, which was later confirmed by phlebography.

A retrospective study conducted with 445 patients (890 limbs), using venous angiotomography to analyze the venous anatomy of patients with varicose veins and edema, found evidence of agenesis of deep femoropopliteal venous segments (as described in this study) in just 0.3% of cases (three limbs).^5^

This condition is one of the causes of CVI, which is currently one of the most frequent diseases affecting both the Brazilian and the global populations. Since this is a chronic and progressive disease, around 3-11% of patients with varicose veins may reach advanced stages at which skin complications are irreversible.^10^ In the case described here, the patient presented with burning pains combined with edema of the limb involved, but did not exhibit clinical signs suggestive of a severe process.

Clinical presentation is variable, ranging from asymptomatic patients to cases with severe venous ulcers.^11^ In the case described, the patient exhibited moderate symptoms of disease unilaterally, with edema and large caliber varicose veins.

Almost all vascular malformations will benefit from correctly prescribed and monitored compressive treatment started as soon as possible, preventing progressive deterioration of clinical status.^12^ In rarer cases in which varicose veins are caused by a congenital venous agenesis, it may be erroneously concluded that the deep vein system is patent and that the case is merely a primary venous insufficiency. However, these cases are problematic when it comes to surgical treatment. If an inappropriate strategy is employed, with removal of the superficial varicose veins, the patient may suffer exacerbation due to overload of the remaining superficial vein system, and it should be remembered that the deep vein system is incomplete.^13^

In cases of hypoplasia or agenesis of the deep vein system, surgical treatment for varicose veins is therefore contraindicated. More appropriate measures include lifestyle changes, wearing elastic stockings, and prescription of palliative medications,^14^ as were adopted in the case described here.

## CONCLUSIONS

Lower limb venous anatomy is highly variable, but agenesis of elements of the deep vein system is rare. Early diagnosis is not always achieved, and the correct therapeutic choice is of fundamental importance in these cases. Management should be founded on conservative approaches and resection of superficial veins should be avoided in these limbs.
